# Dynamic changes of peripheral blood lymphocyte subsets in acute ischemic stroke and prognostic value

**DOI:** 10.1002/brb3.1919

**Published:** 2020-10-27

**Authors:** Jun Xiao, Qian‐Wen Qiu, Chuan Qin, Ran Tao, Su‐Ya Qiao, Man Chen, Deng‐Ji Pan, Dai‐Shi Tian

**Affiliations:** ^1^ Department of Neurology Tongji Hospital Tongji Medical College Huazhong University of Science and Technology Wuhan China

**Keywords:** acute ischemic stroke, lymphocyte subsets, MRS, NIHSS, prognosis

## Abstract

**Objective:**

To explore dynamic changes of peripheral blood lymphocyte subsets in patients with acute ischemic stroke (AIS) and the relationship with stroke severity and long‐term outcomes.

**Methods:**

A total of 96 consecutive patients with AIS and 28 age‐ and gender‐matched healthy controls were recruited. Peripheral blood samples were collected, and the percentages of lymphocyte subsets were analyzed by flow cytometry. The dynamic changes in lymphocyte subsets and their correlation with clinical parameters, such as National Institutes of Health Stroke Scale (NIHSS) scores at onset and modified Rankin scale (mRS) scores 3 months later, were evaluated.

**Results:**

In our study, we observed a decrease in the percentages of T‐lymphocytes (T cells), helper/inducible T‐lymphocytes (Th cells) and suppressor/cytotoxic T‐lymphocytes (Ts cells) in AIS patients as compared to controls. The frequencies of T cells and Ts cells on day 8–14 after stroke in NIHSS ≤4 group were significantly higher than those in NIHSS >4 group. The percentages of T cells and Th cells on day 1–3 after stroke in the mRS ≤2 group were higher than those in the mRS >2 group.

**Conclusion:**

The frequencies of T cells, Th cells, and Ts cells in AIS are declined dramatically at least 14 days after stroke. Lower frequencies of T cells and Ts cells on day 8–14 after stroke represent more severe disease conditions, and the percentages of T cells and Th cells within 72 hr after stroke are negatively correlated with 3‐month outcomes, which might have a potential for predicting long‐term prognosis of stroke.

## Graphical abstract

In this work, we prospectively enrolled patients admitted within 14 days of ischemic stroke onset and investigated the temporal dynamic changes of peripheral blood lymphocyte subsets over different infarction time in relation to clinical severity and prognosis of stroke, aiming to identify the potential prognostic value in patients with AIS.

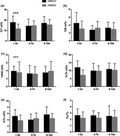

## 1 INTRODUCTION

Stroke is the second leading cause of mortality and morbidity globally and brings huge economic burdens to families and society (Sacco et al., 2013). In China, there are 2.5 million new stroke cases and 1.1 million stroke‐related deaths each year (Liu et al., 2011). Stroke triggers complex immune responses, which play a key role in the pathogenesis of additional brain injury (Becker, 2009; Famakin, 2014). Growing evidence has recently demonstrated that lymphocytes act as mediators and are involved in the development of stroke‐induced immunosuppression (Chamorro & Hallenbeck, 2006; Yan et al., 2009). In the first few hours after stroke, there is a rapid redistribution and activation of lymphocytes, which occurs as part of the peripheral immune response (Liesz et al., 2009). The proportion of T‐lymphocytes has been reported to decrease significantly after AIS (Peterfalvi et al., 2009; Prass et al., 2003; Stubbe et al., 2013), and lymphopenia is correlated with the infarct volume of AIS (Hug et al., 2009).

Dynamic changes of peripheral lymphocyte subsets have been identified as a key predictive element in several central nervous system inflammatory diseases. Patients with multiple sclerosis demonstrate a relationship between the decrease of B and naive CD4^+^ T lymphocytes and neuronal damage indicative of a potential predictor of disease recurrence and disability progression (Posová et al., 2017). Natural Killer (NK) cells can be used as a biomarker for long‐term prognosis after spinal cord injury (Laginha et al., 2016). However, the changes in peripheral lymphocytes at early stage after stroke are contradictory according to several studies (Gill et al., 2018; Laginha et al., 2016; Wang et al., 2017), and the potential relationship between the proportion of peripheral lymphocytes subsets and the severity of acute ischemic stroke remains unclear.

In this work, we enrolled patients admitted within 14 days of ischemic stroke onset and investigated the dynamic changes of peripheral blood lymphocyte subsets over different infarction times in relation to clinical severity and prognosis of stroke, aiming to identify the potential prognostic value in patients with AIS.

## 2 MATERIALS AND METHODS

### 2.1 Subjects

This is a single‐center, observational case–control study of 96 patients with AIS who were admission to Tongji Hospital and 28 age‐ and gender‐matched healthy controls who were recruited from April 2018 to December 2018. Suspected patients with stroke clinical symptoms were confirmed by cerebral CT on admission, and followed by brain MRI. Patients with recent cerebrovascular event (transient ischemia attacks or stroke within 6 months), myocardial infarction (within 3 months), known autoimmune or inflammatory diseases, and acute systemic infections which may influence lymphocytes were excluded. According to the time interval between stroke onset and admission, we divided the patients into three subgroups: 1‐3d group, 4‐7d group, and 8‐14d group. Blood samples were obtained from the subjects between 5:00 a.m. and 6:00 a.m. the next morning after they were hospitalized. The Modified Rankin scale (mRS) scores (range from 0 to 5) were investigated for the purpose to measure the premorbid function of the three subgroups. This study was approved by Ethics Committee of Tongji Hospital (IRB ID: TJ‐C20180312), and written informed consent was obtained from patients or their surrogates before enrollment.

### 2.2 Clinical assessment

The following clinical characteristics were analyzed in the subjects: blood glucose (mmol/L), serum lipid and blood pressure on presentation, cerebral CT and MRI, sex, and age. The stroke severity at onset was evaluated using National Institutes of Health Stroke Scale (NIHSS) ranging from 0 to 42 (Lyden et al., 1994). According to the scale, the severity of stroke was divided into mild (NIHSS score ≤4) and moderate–severe (NIHSS score >4). Patients were followed up by telephone interview at 3 months after stroke, and mRS scores were acquired to measure post‐stroke neurological disability. In general, patients with mRS ≥2 scores are considered to have relatively poor neurological function recovery and various degrees of disabilities. All recording clinicians were certified neurologists and had received suitable training.

### 2.3 Flow cytometry

B cells (CD3 − CD19+), T cells (CD3 + CD19‐), NK cells (CD3‐/CD16 + CD56+), Th cells (CD3 + CD4+), and Ts cells (CD3 + CD8+) were identified and quantified by flow cytometry. The percentages of lymphocyte subsets among total white blood cells were calculated.

### 2.4 Statistical analysis

All data were analyzed by the SPSS Software 24.0 version. Categorical variables were represented as frequencies, which were compared by chi‐square test. Continuous data with normal distribution were expressed as mean ± standard deviation or median (interquartile range, IQR), which were compared by Student's *t* test or one‐way ANOVA. All tests were considered statistically significant at *p* < .05.

## 3 RESULTS

### 3.1 Main characteristics

Ninety six subjects with acute ischemic stroke (age: 58.91 ± 10.05 years; male: female = 2.56:1) were enrolled and 28 volunteers without known cardiovascular cerebrovascular diseases served as controls (age: 54.89 ± 11.04 years; male: female = 1.15:1). There was no significant difference in age (*p* = .072) and gender (*p* = .068) between the two groups. Laboratory and clinical variables of subjects with AIS and control subjects are summarized in Table 1. Of those AIS subjects, 32.3% had medical history of diabetes mellitus and 72.9% had hypertension, the percentage of which was significantly higher than controls. However, the percentage of hyperlipidemia between the two groups had no significant difference, and the total cholesterol, low‐density lipoprotein cholesterol, high‐density lipoprotein cholesterol as well as triglycerides levels in peripheral blood were similar between the stroke patients and controls, which might due to the application of lipid‐lowering medicines. The premorbid mRS scores were 0 in all the subjects.

**TABLE 1:** Demographics and baseline characteristics of AIS patients and controls

	No. (%)		*p* value
	AIS patients (*n* = 96)	Controls (*n* = 28)	
Characteristics			
Age, years	58.91 ± 10.06	54.89 ± 11.04	.072
Sex			
Male	69 (71.9)	15 (53.6)	.068
Female	27 (28.1)	13 (46.4)	.068
Chronic medical illness			
Hypertension	70 (72.9)	13 (42.9)	.003**
Diabetes	31 (32.3)	1 (3.6)	.002**
Hyperlipemia	59 (61.5)	13 (46.4)	.156
Laboratory parameters			
Glycosylated hemoglobin (%)	6.50 ± 1.69	5.52 ± 0.58	.000***
Total cholesterol (mmol/L)	3.85 ± 1.11	3.80 ± 0.80	.822
LDL (mmol/L)	2.44 ± 1.02	2.27 ± 0.63	.281
HDL (mmol/L)	1.00 ± 0.25	1.07 ± 0.25	.229
Triglycerides (mmol/L)	1.55 ± 0.80	1.53 ± 0.87	.893

Note

HDL refers to high‐density lipoprotein, LDL refers to low‐density lipoprotein.

** *p* < .01.

*** *p* < .001.

According to the time interval between stroke onset and admission, the AIS subjects were divided into three subgroups: 1‐3d group (*n* = 31, age: 60.26 ± 11.32 years; male: female = 2.44:1; NIHSS scores: 4 [4]; mRS scores: 1 [1]), 4‐7d group (*n* = 30, age: 58.43 ± 10.51 years; male: female = 2.33:1; NIHSS scores: 3 [3.5]; mRS scores: 1 [1]), and 8‐14d group (*n* = 35, age: 58.11 ± 8.52 years; male: female = 2.89:1; NIHSS scores: 4 [4.5]; mRS scores: 1 [1]), based on the time interval between stroke onset and blood sampling. There was no significant difference in age (*p* = .660), gender (*p* = .921), NIHSS scores (*p* = .572), or mRS scores (*p* = .826) among the three subgroups. The percentage of hypertension (*p* = .231), diabetes mellitus (*p* = .244), and hyperlipidemia (*p* = .972) had no significant difference, and the levels of high‐density lipoprotein cholesterol and triglycerides were similar among these subgroups. However, the differences in low‐density lipoprotein cholesterol (*p* = .001) and total cholesterol (*p* < .001) among subgroups were statistically significant. Further analysis showed that 8‐14d group had significantly lower values of the total cholesterol and low‐density lipoprotein cholesterol than 1‐3d group and the 4‐7d group, while there was no statistical significance between the 1‐3d group and the 4‐7d group, which may be attributed to the use of statin agents. Acute treatment (IV alteplase, 0.9 mg/kg, maximum dose 90 mg over 60 min with initial 10% of dose fiven as bolus over 1 min) was received in 2 subjects in 1‐3d group, 3 in 4‐7d group, and 3 in 8‐14d group, respectively. There was no significant difference in acute treatment among the three subgroups (*p* = 1.00). Meanwhile, dual antiplatelet therapy (aspirin, 100 mg/d and clopidogrel, 75 mg/d) was taken in all subjects. No patient was undertaken mechanical thrombectomy.

### 3.2 Changes in the peripheral lymphocyte subsets after AIS

**FIGURE 1:**
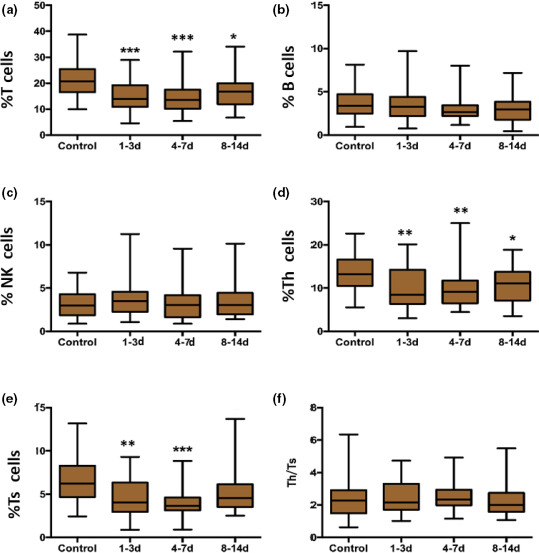
Temporal change of lymphocyte subsets in AIS patients by flow cytometry in AIS patients. Notes: % T cells = T‐cell count/leukocyte count, % B cells = B‐cell count/leukocyte count, % NK cells = NK‐cell count/leukocyte count, %Th cells = Th‐cell count/leukocyte count, %Ts cells = Ts‐cell count/leukocyte count. *p < .05, **p < .01 and ***p < .001

The number of T cells, Th cells, and Ts cells in peripheral blood was detected using flow cytometry. The population was expressed as percentage (lymphocyte count/leukocyte count). There was a general downward trend in the percentage of the above mentioned lymphocyte subsets among total leukocytes in comparison to the control group. Compared with controls, the decline in the percentages of T cells, Th cells, and Ts cells reached statistical significance in all the subgroups. There was a dramatic loss of T cells (*p* = .001), Th cells (*p* = .005), and Ts cells (*p* = .009) from 1 to 3 days, the values decreased reaching its nadir on 4–7 days (*p* < .01), and the percentages of which on 8–14 days showed slight recovery ( Figure 1a,d,e). On the other hand, there was a slight increase in the percentage of NK cells, a slight decrease in the percentages of B‐lymphocyte and Th/Ts ratio in the peripheral blood of stroke patients in all the three subgroups compared with controls, but not statistically significant ( Figure 1b,c,f).

### 3.3 Association between the percentages of lymphocyte subsets and clinical severity of AIS

**FIGURE 2:**
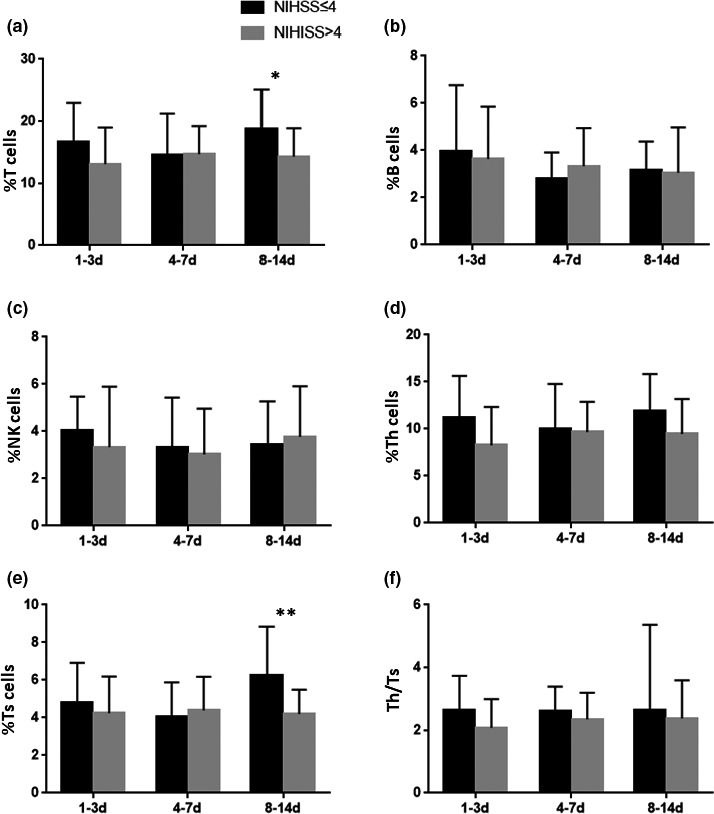
The relationship between temporal change of lymphocyte subsets and the severity in AIS patients. The percentages of T cells and Th cells on 8–14 group in NIHSS ≤4 group were significantly higher than those in NIHSS >4 group. Notes: % T cells = T‐cell count/leukocyte count, % B cells = B‐cell count/leukocyte count, % NK cells = NK‐cell count/leukocyte count, %Th cells = Th‐cell count/leukocyte count, %Ts cells = Ts‐cell count/leukocyte count. *p < .05, **p < .01

To investigate the association between the percentages of lymphocyte subsets and NIHSS scores, patients in the AIS group were divided into NIHSS ≤4 group (mild stroke, *n* = 53) and NIHSS >4 group (moderate and severe stroke, *n* = 43). The percentages of T cells (*p* = .023) and Ts cells (*p* = .007) on day 8–14 after stroke in NIHSS ≤4 group were significantly higher than those in NIHSS >4 group, showing statistical significance (see Figure 2a,e), while no significant difference was found between NIHSS ≤4 and NIHSS >4 groups with regard to the percentages of B‐lymphocyte, NK cells, Th cells, and Th/Ts ratio (see Figure 2‐d,f).

### 3.4 Correlation between the percentages of lymphocyte subsets and the 3‐month prognosis after AIS

**FIGURE 3:**
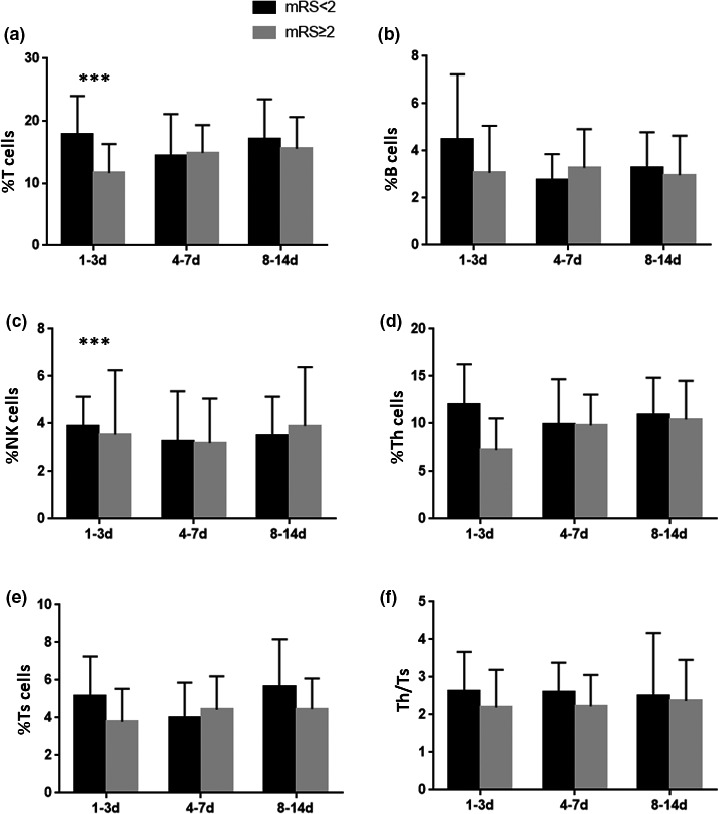
The relationship between temporal change of lymphocyte subsets and the outcome at 3 months in AIS patients. Notes: % T cells = T‐cell count/leukocyte count, % B cells = B‐cell count/leukocyte count, % NK cells = NK‐cell count/leukocyte count, %Th cells = Th‐cell count/leukocyte count, %Ts cells = Ts‐cell count/leukocyte count. **p < .01

To determine possible predictive value of stroke outcomes, the patients were divided into two subgroups based on mRS scores at 3 months after stroke: mRS <2 group (excellent outcome, *n* = 58) and mRS ≥2 group (good or less outcome, *n* = 38). As shown in Figure 3, the percentages of T cells and Th cells on day 1–3 after stroke in the mRS <2 group were higher than those in the mRS ≥2 group (for T cells: *p* = .005; for Th cells: *p* = .002, Figure 3a,c). The percentages of Ts cells, B cells, NK cells, and Th/Ts ratio showed no significant difference between mRS <2 group and mRS ≥2 group (see Figure 3b,d‐f).

## 4 DISCUSSION

Stroke has enormous effects on the normally well‐balanced interplay of the two super systems: the nervous system and the immune system. Recent studies elucidated that immunosuppression can be detected within a few hours after ischemia, and lasts for several weeks. Stroke‐induced immunosuppression may have beneficial effects and is highly relevant for functional outcome after stroke (Dirnagl, 2004; Liu et al., 2017). In particular, emerging evidence has demonstrated that lymphocytes play an important role in the immunosuppression after stroke (Vogelgesang et al., 2010; Yan et al., 2009; Yilmaz et al., 2006). The reduction in number and functions of circulating lymphocytes was also associated with the lower mortality and better outcome of experimental stroke (Chu et al., 2014; Hurn et al., 2007; Schwartz & Moalem, 2001). However, clinical research on this issue has yielded conflicting results (Noh et al., 2018; Vogelgesang et al., 2008; Wang et al., 2017; Yan et al., 2009). Some research has indicated that a profound infiltration of inflammatory cells (monocytes, macrophages, B lymphocytes, Ts cells, and NK cells) occurs in the brain early after focal ischemia (3 hr after stroke), especially without reperfusion(Gendron et al., 2002), while others showed that the percentages of activated T cells and regulatory T cells were significantly increased in patients with ischemic stroke compared to healthy subjects(Yan et al., 2009). Thus, the changes in blood lymphocyte subsets at different stages after ischemic stroke remain controversial.

In line with previous reports (Wang et al., 2017; Yan et al., 2009), we observed a dramatic decrease in the percentages of T cells in the peripheral blood of patients within 14 days of stroke onset, compared to age‐ and sex‐matched controls. We also found a rapid loss of Th cells and Ts cells from peripheral blood of patients with stroke, suggesting that defects of Th‐ and Ts‐cell function contribute to immunosuppression in stroke. These results support the notion that T cells participate in the stroke‐induced immunosuppression in humans, which causes a rapid and sustained decline of T cells, Th cells, and Ts cells in peripheral blood. Besides, previous studies have shown that T‐lymphocyte deficiency mice had smaller infarct volume in various models of acute ischemia stroke (Kleinschnitz et al., 2010; Liesz et al., 2011; Stubbe et al., 2013; Yilmaz et al., 2006). These lymphocytes are known to play an important role in the acute phase of ischemic stroke, but the mechanism has not been clarified. One of the possible mechanisms is that the sympathetic nervous system and the hypothalamus–pituitary–adrenal axis function are highly active, leading to the elevated levels of glucocorticoid and katecholamina hormones, causing the decrease of spleen cells, making spleen atrophy and apoptosis of the immune cells in spleen, eventually resulting in a decrease of peripheral blood lymphocytes(Kohm & Sanders, 2001).

Further analysis has been made to reveal the possible relationship between the populations of lymphocyte subsets and stroke severity. NIHSS score is a commonly used indicator to evaluate stroke severity. We detected a negative correlation between the percentages of NIHSS scores and the percentage of lymphocytes. In specific, we first found that the frequencies of T cells and Ts cells on day 8–14 after stroke in NIHSS ≤4 group were significantly higher than those in NIHSS >4 group, indicating that lower frequencies of T cells and Ts cells on day 8–14 after stroke represent more severe stroke conditions.

Besides, this study has also revealed that the percentages of T cells and Th cells in 1‐3d group after stroke in subjects with excellent outcome were higher than those in subjects with good or less outcome, showing that the mRS scores at 3 month after stroke were negatively correlated with the percentages T cells and Th cells within 72 hr after stroke. These data, for the first time, suggest a potential value of T and Th cells within 72 hr after stroke to predict long‐term recovery after stroke, which might be used as an indicator for prognosis of ischemic stroke.

The post‐stroke changes in B cells, NK cells, and Th/Ts ratio remain elusive due to contradictory results. Th/Ts ratio is an indicator to the immune disorder, decreasing of which indicates the immunosuppressive state, commonly seen in immunodeficiency diseases. NK cells can directly kill target cells without relying on antibodies and complement, and also have immunomodulation function, involved in some autoimmune diseases. More and more studies have revealed that NK cells have potential functions in bacterial defense, participating in immunosuppression of stroke and infections afterward (Klehmet et al., 2009; De Raedt et al., 2015). Some studies observed reduction of B cells, NK cells, and Th/Ts ratio after stroke (Hug et al., 2009; Wang et al., 2017), while others reported unchanged or even elevated cell counts in stroke patients (Haeusler et al., 2008; Klehmet et al., 2009; Tuttolomondo et al., 2015; Vogelgesang et al., 2010). In our study, the percentage of B cells and NK cells and Th/Ts ratio did not show significant difference as compared to controls, and there was no significantly correlation between B cell, NK cell proportion, and stroke severity or outcomes. More studies are warranted in the future to elucidate these discrepancies.

## 5 CONCLUSION

The present study suggests a rapid and relatively sustained decline in T cells, Th cells, and Ts cells after acute ischemic stroke and provides evidence of negative correlation between T cells, Ts cells on day 8–14 after stroke and stroke severity. T cells and Th cells within 72 hr after stroke are negatively correlated with long‐term outcomes, which might be promising biomarkers for predicting 3‐month prognosis. There are several limitations in this study. Some lymphocyte types, such as regulatory T cells, are not included in our study. Besides, each patient was a single data point, not sampled multiple times and then assumptions were made about trends in lymphocyte counts at three subgroups. There is no temporal data of lymphocyte counts for the stroke subjects in our study. Despite the limitation, we have considered various confounding effects, such as age, sex, the premorbid function, the severity of stroke, the use of IV alteplase, and antiplatelet agents, which have no significant difference among the three subgroups, making the conclusions of the manuscript more reliable. Larger scale and long‐term prospective trials are needed to confirm the correlation between the peripheral lymphocyte responses and the stroke outcomes and may possibly establish biomarkers to predict long‐term prognosis.

## AUTHOR CONTRIBUTION

Jun Xiao, Chuan Qin, Deng‐Ji Pan, and Dai‐Shi Tian were responsible for designing the study; Qian‐Wen Qiu, Ran Tao, Su‐Ya Qiao, and Man Chen were responsible for acquiring and analyzing the clinical data; Jun Xiao, Qian‐Wen Qiu, Chuan Qin, and Dai‐Shi Tian were responsible for drafting the manuscript and figures. All the authors reviewed, revised, and approved the final manuscript.

### Peer Review

The peer review history for this article is available at https://publons.com/publon/10.1002/brb3.1919.

## Data Availability

The data that support the findings of this study are available from the corresponding author upon reasonable request.
